# Supplementary material for: Chromosomal Distribution of Cytonuclear Genes in a Dioecious Plant with Sex Chromosomes
Source: Genome Biol Evol. 2014 Sep 4;6(9):2439–43. doi: 10.1093/gbe/evu197 (PMC4202333; doi:10.1093/gbe/evu197)
Supplement: Supplementary Data [file supp_6_9_2439__index.html]

Chromosomal Distribution of Cytonuclear Genes in a Dioecious Plant with Sex Chromosomes — Supplementary Data 

# Chromosomal Distribution of Cytonuclear Genes in a Dioecious Plant with Sex Chromosomes

## Supplementary Data

files

**Files in this Data Supplement:**

- Supplementary Data - docx file
