# Supplementary material for: Chromosomal Distribution of Cytonuclear Genes in a Dioecious Plant with Sex Chromosomes
Source: Genome Biol Evol. 2014 Sep 4;6(9):2439–43. doi: 10.1093/gbe/evu197 (PMC4202333; doi:10.1093/gbe/evu197)
Supplement: Supplementary Data [file supp_evu197_supp_Aug14.docx]

**SUPPLEMENTARY MATERIAL**

**Chromosomal distribution of cyto-nuclear genes in a dioecious plant with sex chromosomes**

Josh Hough^*†^, J. Arvid Ågren^*§^, Spencer C.H. Barrett, and Stephen I. Wright

Department of Ecology and Evolutionary Biology, University of Toronto, Toronto, ON, Canada, M5S 3B2

**Power Analysis**

To determine the extent to which a biased distribution of cyto-nuclear genes could be detected given our sample sizes of annotated autosomal, X-linked, and X-hemizygous genes, we calculated the power to detect significant differences based on a Fisher’s Exact Test. Here, power refers to the probability of correctly rejecting the null hypothesis of no difference in the proportion of cyto-nuclear genes among the gene sets, and we used the hypergeometric distribution to calculate the probability of getting the observed data under the null hypothesis that the proportions were the same (with an alpha significance level of 0.05).

To better visualize the difference in power for the two main comparisons of interest (autosomal genes vs. X/Y genes, and autosomal genes vs. X-hemizygous genes), the figure below shows an example in which the true proportion of cyto-nuclear genes on autosomes is assumed to be 0.25 (which is approximately the empirical proportion in our data). Power is then shown as a function of the true proportion of cyto-nuclear genes on the X-chromosome, ranging from 0.1 to 0.5. As discussed in the main text, our sample sizes of annotated X/Y genes (n=567; see Table 1 in main text) were large enough to detect differences between autosomes and X/Y genes with ~80% power given a true difference of ~5%, and as this difference becomes smaller, the power decreases. Similarly, for hemizygous X-linked genes (with n=95), differences of approximately~10% could be detected with ~80% power, and power was reduced for smaller differences. This analysis was done using G*Power (Faul et al. 2007) and R (R Development Core Team 2013).


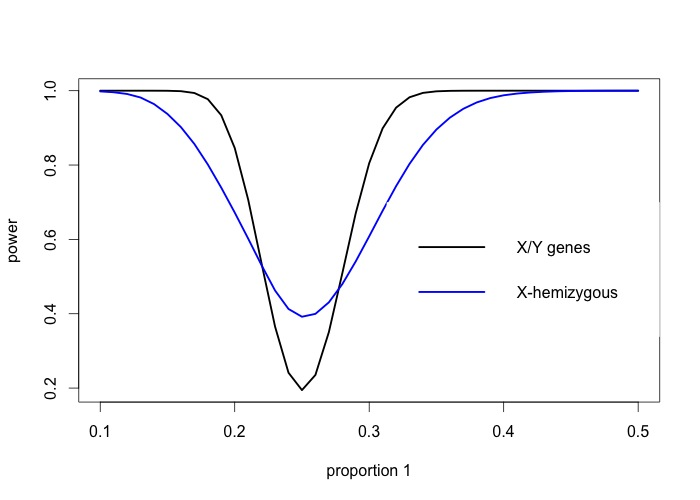


Figure 1- Power to detect a significant difference in the proportion of cytoplasmic genes between autosomes and sex chromosomes as a function of the true proportion on sex chromosomes. Power was calculated assuming the true proportion of cytoplasmic genes on autosomes was 25%.

**References**

Faul F, Erdfelder E, Lang A.-G., and Buchner A. 2007. G*Power 3: A flexible statistical power analysis program for the social, behavioral, and biomedical sciences. Behavior Research Methods 39:175–191

R Development Core Team. 2013. R: A language and environment for statistical computing. Vienna, Austria: R Foundation for Statistical Computing.
